# Collagen fleece grafting for surgical treatment of patients with mild to severe peyronie’s curvatures

**DOI:** 10.1007/s11255-024-04222-2

**Published:** 2024-10-08

**Authors:** Radion Garaz, Bastian Amend, Arnulf Stenzl, Jens Bedke, Jörg Hennenlotter, Alexander Rochwarger, Christian M. Schürch, Igor Tsaur, Steffen Rausch

**Affiliations:** 1 Department of Urology, Eberhard Karls University of Tübingen, Hoppe-Seyler-Straße 3, 72076 Tübingen, Germany; 2 Department of Urology and Transplantation Surgery, Stuttgart Hospital, Stuttgart, Germany; 3 Department of Pathology and Neuropathology, Eberhard Karls University of Tübingen, Tübingen, Germany

**Keywords:** Grafting, TachoSil, Induratio penis plastica, Plaque incision

## Abstract

**Purpose:**

Collagen fleece grafting (CFG) is the recommended treatment for severe Peyronie’s disease (PD) curvature (> 60°), but its efficacy in mild/moderate curvatures remains uncertain. This study evaluated CFG in patients with mild/moderate curvatures (< 60°) at risk of penile shortening or symptomatic plaque.

**Methods:**

A retrospective review was conducted on patients who underwent surgical treatment for PD using plaque incision or partial plaque excision and CFG. Clinical parameters and complications were reviewed. Subgroup analysis was performed on patients with curvatures of > 60° and curvatures ≤ 60°.

**Results:**

89 patients with a median age of 59 years and a median curvature of 70 (20–90)° were identified. Dorsal curvature was predominant in 66% of cases, followed by lateral (16%), ventral (8%), and complex curvatures (10%). Partial plaque excision was performed in 98% of patients, with an average grafting area of 2.1 cm^2^; 71% had a singular penile plaque, while 29% presented two or more plaques. The comparison between patients with curvatures ≤ 60° and > 60° revealed no significant differences in mean operation time (86.3 vs. 94.4 min, *p* = 0.13) or in the incidence of postoperative complications, including glans necrosis, hypoesthesia, ecchymosis, bleeding, hematoma, infection, residual curvature, revision surgery, or pain.

**Conclusions:**

Early postoperative outcomes and complication rates following plaque incision or partial plaque excision and grafting with CFG were comparable in patients with mild/moderate and severe PD deformities. The technique may be a viable option with a similar risk profile for achieving penile straightening in selected PD cases, particularly when plication is not feasible.

## Introduction

Peyronie’s disease (PD) is an underestimated condition with estimated prevalence rates ranging from 0.4 to 20.3% [1]. Typically, males between 40 and 70 years of age are affected. The curvature, loss of penile length, and erectile dysfunction (ED) associated with PD may be highly symptomatic, causing emotional, sexual, and psychological distress [2]. The natural history of PD includes the acute (inflammatory) and chronic (fibrotic) stages [3].

Spontaneous resolution of the penile plaque and curvature can occur in a minority of PD cases, likely less than 10% [3]. Due to the overall limited level of evidence, definitive recommendations for the optimal treatment of PD have not been firmly established [3–6]. Conservative therapy is typically used in the active phase of PD to stabilize the disorder, preventing further curvature progression and shortening [7]. Currently, penile straightening surgery represents the gold standard for treating penile deformity during the stable phase without ED due to its high efficacy and low morbidity [6]. Recommended surgical techniques for PD therapy can be categorized into three main groups: (1) tunical shortening procedure for patients with curvature less than 60 degrees, good erectile function, adequate penile length, and no complex deformities; (2) tunical lengthening procedure involving grafting techniques after plaque incision (PIG) or partial plaque excision (PEG) for patients with severe curvature, complex deformity, and reduced penile length; and (3) penile prosthesis implantation (PPI) with or without straightening techniques for patients with concomitant ED unresponsive to conservative therapy and residual curvature [3, 6, 8, 9].

Grafting techniques are recommended as the surgical treatment of PD for patients in the stable stage exhibiting severe penile curvature, shortened penis, hourglass deformity, or the hinge effect [9]. However, the definition of severe curvature typically suggested to be greater than 60 degrees, lacks validation through empirical studies. In addition, candidates for grafting without a penile prosthesis should display good preoperative erectile function, as there is a significant risk of postoperative worsening of erectile function [3]. It is important to note that none of the currently available graft options fully meet the criteria for an ideal graft in the surgical repair of PD. Nevertheless, there is a growing inclination toward xenografts, such as porcine small intestine submucosa (SIS), bovine pericardium, or collagen fleece [5, 10]. Among these options, grafting with collagen fleece (CFG) xenograft (TachoSil) shows promise due to its effectiveness, safety, practicality, and ease of use, which contribute to reduced operating times and costs [5, 10, 11]. Despite the available information, reconstructive urology surgeons require further guidance to select the most suitable graft for their patients [4].

Penile plication is typically recommended for patients with mild to moderate curvature in PD, while grafting techniques in these cases have been reported infrequently [6]. Plication techniques may not always be adequate, especially in PD with deviations < 60°, particularly when there are issues such as reduced penile length or complex deformities [12]. The present study aimed to assess the surgical outcomes of PIG/PEG and grafting with CFG in such patients.

Specifically, we aim to compare the clinical and surgical results of men treated with these techniques for mild/moderate curvature (group A—less than 60°) with those with severe curvature (group B—more than 60°).

## Materials and methods

### Study design, ethics, and patients

This study was a retrospective review of patients with PD who underwent surgical treatment from January 2012 to June 2022 at the University Hospital of Tübingen, Germany. During this period, 122 PD patients in the stable phase underwent surgical intervention. The surgical indications were a PD duration ranging from at least 12 months from onset, previous conservative therapies failed, and at least 6 months of quiescent stage (without pain or deformity deterioration) to minimize the risk of subsequent disease progression and recurrence of the curvature. Patients who needed preoperative phosphodiesterase type 5 inhibitors (PDE5i) or intracavernous injections were also included in the analysis. Patients with severe ED who did not respond to medical treatment or vacuum pump therapy and needed PPI were excluded from the study. Patients who had undergone previous surgeries for PD were not excluded from the study. For CFG, collagen fleece grafts (TachoSil; Corza Medical; Switzerland) were applied. Patients were excluded if they received grafting with materials other than collagen fleece grafts or other surgical procedures (e.g., plication procedures and PPI). The potential benefits and risks of surgery were thoroughly discussed with each patient before surgery. All data used in this study were collected retrospectively from the patients’ medical records. The study was approved by the local Ethics Committee at the University of Tübingen (Approval Number 389/2008BO2 [13]).

### Preoperative evaluation

The preoperative assessment included a detailed medical and sexual history, a physical examination, and a penile duplex ultrasound. Plaque localization was determined by penis palpation and ultrasonography using a 7.5 MHz linear transducer. Plaque size was measured with ultrasound. In addition, the degree of curvature was documented preoperatively with self-photography of the penis at maximum erection, from above laterally and frontally, and, if considered necessary, an artificial erection was pharmacologically induced [14].

Before surgery, the erectile function was assessed by the International Index of Erectile Function Questionnaire (IIEF-5-Score). Scores below 16 points were defined as low to moderate ED. Age, hypertension, hypogonadism, hyperlipidemia, diabetes mellitus (DM), Dupuytren’s disease, nicotine abuse, alcohol abuse, or previous prostate operation (transperitoneal radical prostatectomy or transurethral resection of the prostate) were recorded.

### Surgical procedure

All surgeries were performed under general anesthesia. In total, four different trained surgeons performed the interventions. A transurethral catheter was placed intraoperatively. In all cases, the degree of curvature and localization was documented again. We injected a physiological saline solution (NaCl) into the corpora, thus inducing an artificial erection. During the intervention, we performed circumcision unless it had been done before. Then, the penile degloving on Buck’s fascia was performed, and an artificial erection was induced again to identify the precise position of the maximum curvature point. Magnifying glasses were used to mobilize the dorsal neurovascular bundle (NVB).

The surgical technique was selected based on the patient’s preference, degree of penile curvature, penile length, and erectile function. The preferred approach for lengthening procedures (LP) was partial plaque excision or incision and addition grafting with CF [15]. A plaque incision was done by a simple transverse incision of the tunica albuginea (TA). Occasionally, several incisions were made. Partial plaque excision was performed by excising an ellipsoid part of the TA at the maximum curvature point. Subsequently, the incision or partial excision site was covered with a TachoSil patch according to the manufacturer’s directions without any sutures. Care was taken to overlap the patch and intact tunica by about 1 cm. The hydraulic test was not conducted following the placement of TachoSil. The neurovascular bundle was re-approximated after manual compression for at least three minutes, and Buck’s fascia was closed. Then, the penile skin was closed, and a compression dressing was applied. We did not insert surgical drainage at the end of the surgeries.

### Postoperative follow-up

A wound control was performed about 3 h postoperative to check for bleeding and sensitivity of the penile glans. Postoperatively, patients without evidence of ED were administered diazepam for 7 days to prevent physiological erection at night. All patients were advised to refrain from sexual activity for 6 weeks postoperatively.

### Outcome measures

Hospital stays, antibiotic therapy after discharge, catheter placement, compress dressing, and surgical recurrence (operative time, intraoperative complications) were recorded. The outcome was assessed during early clinical follow-up. The postoperative follow-up period was a maximum of 6 months. We evaluated the outcomes in terms of curvature correction, penile length, erectile function, patient satisfaction, and postoperative complications.

### Statistical analysis

For comparison, two groups (A. mild/moderate curvature < 60, B. severe curvature > 60) were created to assess the association of clinical or surgical outcomes with severe curvatures. Continuous variables are presented with the mean, SD, and range. The Shapiro–Wilk test was used to assess the Normal distribution. Student’s t-test, Fischer’s exact test or a Chi-square test, and the Mann–Whitney *U* test were used to compare continuous and categorical variables between the subgroups, respectively. A *p* value of < 0.05 was considered statistically significant*.* All statistical analyses were performed using GraphPad Prism, v.8 (GraphPad Software, San Diego, California, USA).

## Results

### Patient sample and preoperative condition

Of 122 patients who underwent surgical treatment for PD, 89 (73%) male patients underwent PIG or PEG and grafting with a TachoSil patch during the study period and were included in this retrospective analysis. The patients’ mean (range) age at the time of the surgery was 57.8 (38–73) years. The direction of the curvature was dorsal in 59 patients (66%), ventral in 7 (8%), lateral in 14 (16%), and complex in 9 (10%). The primary curvature’s mean (SD) angle was 68 (18)°. Before surgery, 54 patients (61%) had > 60° curvature and 34 patients (39%) had 30–60° curvature. Two patients had undergone previous surgical treatment for PD. Based on the patients’ reports, 26% had mild to moderate ED preoperatively (Table 1).

**Table 1:** Patients’ characteristics

Number of patients (n)	89
Age, years, mean ± SD (range)	57.81 ± 6.76 (38‒73)
< 40 years (n)	1
Comorbidities	
Dupuytren’s disease, n (%)	7 (7.87)
Hypertension, n (%)	22 (24.72)
Hyperlipidemia, n (%)	2 (2.25)
DM*, n (%)	9 (10.11)
Hypogonadism, n (%)	2 (2.25)
Nicotine abuse, n (%)	8 (8.99)
Previous prostatectomy, n (%)	3 (3.37)
Previous TURP, n (%)	4 (4.49)
Idiopathic curvature (n)	85
Post-traumatic curvature (n)	4
Previous treatment	
No treatment (n)	65
POTABA* therapy (n)	13
Vitamin E (n)	7
Glucocorticoids injections (n)	1
ESWT* (n)	1
Surgical treatment (n)	2
Preoperative ED, n (%)	23 (25.84)
Preoperative IIEF-5* score, mean ± SD (range)	19.25 ± 4.08 (9‒25)

*DM diabetes mellitus, *TURP* transurethral resection of the prostate, *POTABA* potassium paraaminobenzoate, *ESWT* extracorporeal shockwave treatment, *ED* erectile dysfunction, *IIEF-5 Score* international index of erectile function questionnaire

### Surgical management and intraoperative outcomes

**Fig. 1:**
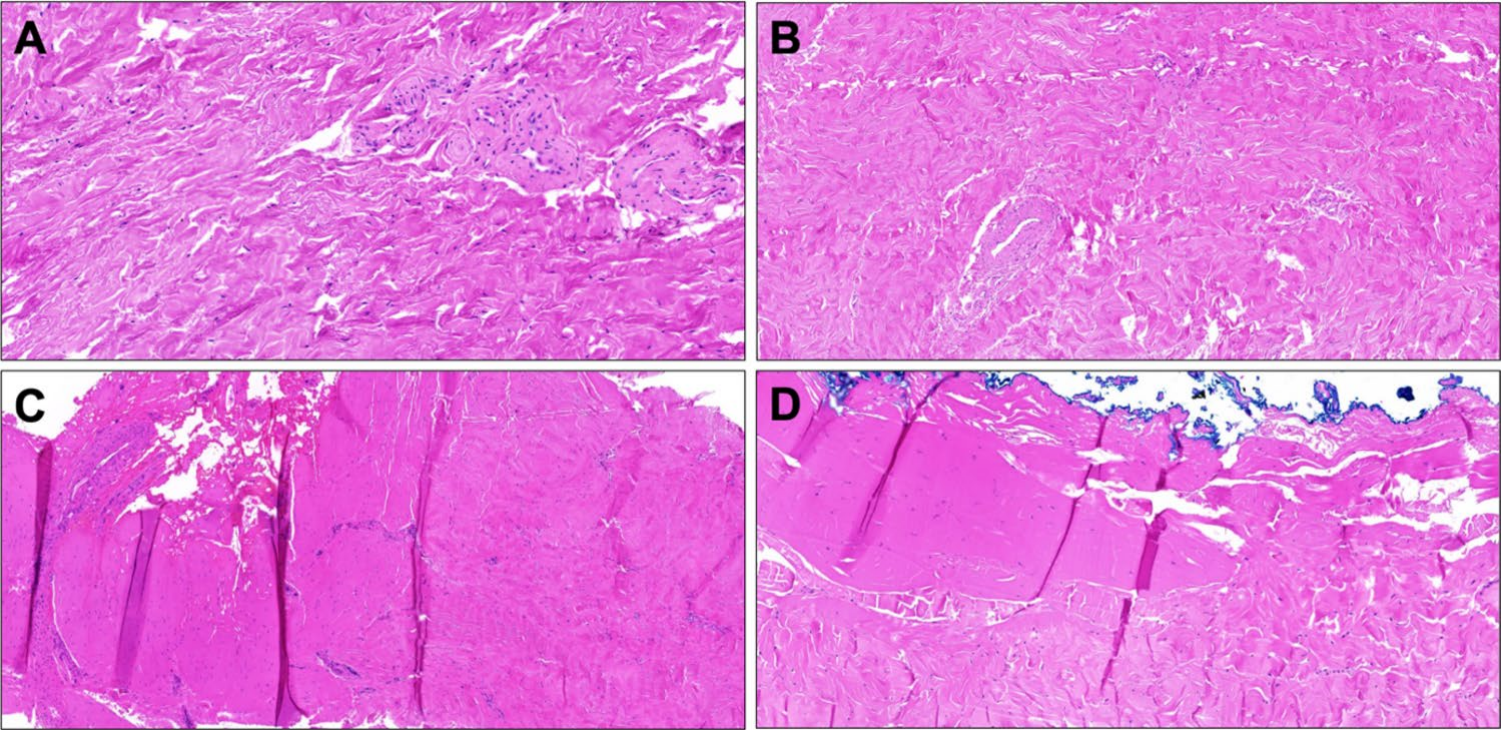
Histological presentation of IPP plaque. **A** Scar tissue with lamellar fibrosis and prominent vessels; **B** Paucicellular fibrosis presenting with lamellar organization, no inflammation, no ossification; **C** scar tissue with nodular fibrosis and prominent vessels with increased perivascular cellularity; **D** paucicellular fibrosis shows transient organization as dense fibrosis (near resection, marked blue) is followed by lamellae. Hematoxylin–eosin staining, 25 × magnification

The number of procedures performed by the four surgeons was 12, 15, 30, and 32, respectively. In three patients, additional plication with Polydioxanone 2–0 suture was performed (Essed–Schroeder method) to correct a persisting concomitant lateral deviation, aiming to achieve straightness. Additional plication (Nesbit technique) was performed in one case. Histologically, the excised plaques showed no evidence of malignancy (Fig. 1). The mean (SD) operative time was 91 (21) min. The mean (range) hospital stay was 5.1 (3‒11) days. Postoperative catheterization was performed in 77 of 89 patients. After being discharged, 11 patients (12%) received antibiotic therapy for prophylactic reasons (Table 2).

**Table 2:** Intra-operative and early postoperative outcomes

Penile curvature, mean ± SD (range)	68 ± 18° (20°‒90°)
< 30°, n (%)	1 (1.12)
30°‒60°, n (%)	34 (38.21)
> 60°, n (%)	54 (60.67)
Secondary curvature, mean ± SD (range)	20 ± 8.2° (10°‒30°)
Direction of curvature	
Ventral, n (%)	7 (7.87)
Dorsal, n (%)	59 (66.29)
Lateral (left or right), n (%)	14 (15.73)
Complex (lateral and dorsal/ventral), n (%)	9 (10.11)
Mean operative time ± SD, minutes (range)	91 ± 21 (49‒152)
Number of penile plaques	
One, n (%)	63 (70.79)
Multiple (≥ 2), n (%)	26 (29.21)
Partial plaque excision, n (%)	87 (97.75)
Plaque incision, n (%)	2 (2.25)
Mean surface area of grafting ± SD, cm^2^ (range)	2.1 ± 1.9 (0.13‒12.8)
Procedure	
Plaque excision/incision and grafting with TachoSil, n (%)	85 (95.51)
Nesbit and grafting with TachoSil, n (%)	1 (1.12)
Essed–Schroeder and grafting with TachoSil, n (%)	3 (3.37)
Concomitant circumcision, n (%)	52 (58.43)
Hospital stays, mean ± SD, days (range)	5.1 ± 1.2 (3‒11)
Postoperative catheterization	
Yes (n)	77
No (n)	12
Postoperative catheterization, mean ± SD, days (range)	2.3 ± 1.3 (1‒6)
Postoperative antibiotic treatment, n (%)	11 (12.36)
Postoperative Diazepam treatment, n (%)	37 (41.57)
Compression dressing, mean ± SD days (range)	3.98 ± 0.53 (2‒6)

### Postoperative complications

Surgical complications are summarized in Table 3.

**Table 3:** Early postoperative complications

Complications	
Ecchymosis, n (%)	10 (11.24)
Hematoma, n (%)	4 (4.49)
Glans necrosis, n (%)	1 (1.12)
Bleeding, n (%)	3 (3.37)
Wound infection, n (%)	2 (2.25)
Pain on graft side, n (%)	5 (5.62)
Decreased sensation in the penis glans, n (%)	8 (8.89)
Residual curvature, n (%)	5 (5.62)
Curvature recurrence, n (%)	8 (8.99)
None, n (%)	33 (37.07)

### Clinical associations with the severity of the curvature

There was no statistically significant difference between the comorbidities of the patient groups except for DM, which was associated with more severe curvature (*p* = 0.01). Preoperative ED was also more frequent in the > 60° group (11% vs. 35%, *p* < 0.0001). There was no difference between the groups regarding hospital stay, operating time, or surgical complications (Table 4).

**Table 4:** Comparison between mild/moderate to severe curvature

	Curvature ≤ 60° (35)	Curvature > 60° (54)	*P* value	95% CI*	OR*
Age, y, mean ± SD (range)	58.11 ± 8.16 (41–72)	56.95 ± 8.60 (38–73)	0.66	− 4.255 to 6.569	
Comorbidities					
DD*	1	6	0.23	0.0199–1.595	0.23
Hypertension	6	16	0.21	0.1684–1.393	0.49
Hyperlipidemia	0	2	0.51	0.000 to 3.328	0.000
DM*	0	9	**0.01**	0.000 to 0.5532	0.000
Hypogonadism	0	2	0.51	0.000–3.328	0.000
Nicotine abuse	4	4	0.70	0.4410 to 5.836	1.61
Prostatectomy	2	1	0.56	0.3686–48.76	3.13
TURP*	2	2	0.63	0.2439 to 10.71	1.62
Hospital stays, days	5.09 ± 1.15	5.09 ± 1.32	0.76		
Operation time	87.5 ± 17.4	93.3 ± 23.0	0.20	− 3.194 to 14.910.48	…
Preoperative treatment	26%	28%	0.75	60–1.666	0.90
Preoperative ED*	11%	35%	** < 0.0001**	0.1087–0.4905	0.24
Complications					
Ecchymosis	5	5	0.50	0.4715 to 5.617	1.63
Hematoma	1	3	> 0.99	0.0375 to 3.496	0.50
Bleeding	2	1	0.55	0.3578 to 47.29	3.21
Wound infection	1	1	> 0.99	0.0798 to 30.11	1.55
Postoperative antibiotics	6%	17%	0.11		
Pain on the graft side	3	2	0.37	0.4720 to 14.16	2.43
Glans hypoesthesia	3	5	> 0.99	0.2308 to 3.880	0.91
Residual curvature	3	2	0.37	0.4720 to 14.16	2.43
Curvature recurrence	2	6	0.47	0.0955 to 2.115	0.48

*CI confidence interval, *OR* odds ratio, *DD* Dupuytren’s disease, *DM* diabetes mellitus, *TURP* transurethral resection of the prostate, *ED* erectile dysfunction

## Discussion

The success of PD reconstruction is defined by a postoperative penile curvature of less than 20 degrees, preservation of erectile function, and the ability to engage in sexual intercourse [6]. However, reduced penile length and discomfort or pain caused by extensive plaque may present additional functional challenges in PD, requiring specific surgical strategies. Two potential surgical strategies for penile lengthening in severe PD cases include (1) PIG or PEG without PPI implantation and (2) PPI with or without simultaneous TA incision and grafting [4, 8].

Various grafts have been used to close the tunica albuginea defect following plaque incision or partial plaque excision, including autologous grafts, allografts/xenografts (tissue engineering grafts), and synthetic grafts. An ideal graft should be readily available, resistant to infections, promote hemostasis, minimize postoperative contracture, and be cost-effective [10, 16]. Most synthetic grafts have been discarded due to fibrotic tissue reactions, graft contracture, allergic reactions, and an increased risk of infection [5]. Xenografts are preferred because they offer better outcomes, lack donor site morbidity, and require less operating time than autografts [16]. Currently, the small intestine submucosa is one of the most frequently used xenografts in PD surgery [6].

A technique involving partial plaque excision and grafting with collagen fleece coated with tissue sealant (TachoSil; Mycomed; Konstanz, Germany) has been described [15]. The collagen fleece includes a fibrin glue coating, which precludes the need for fixation or suturing and provides a hemostatic effect. Grafting with collagen fleece requires less operating time because fixation is not necessary. However, more extensive incisional techniques to the tunica albuginea, the application of the patch without fixation, and the mobilization of the NVB for PIG/PEG are surgical aspects that might influence the perioperative and postoperative course of the patients. This raises the question of whether the technique should be reserved for cases with curvatures > 60°, given that the usually less invasive plication procedures are recommended for curvatures < 60° by most guidelines.

Our study identified a significant association between DM and severe curvature; the number of patients with DM was significantly higher in the severe curvature subgroup (> 60°). However, both subgroups were similar in terms of age, hospital stay, operative time, early results, and complications. In addition, we found no significant differences in operating time or early postoperative complications between the two subgroups with curvatures > 60° and < 60°, indicating that the technique can be applied with the same risk profile regardless of the degree of deviation.

After performing PEG and CFG in 63 men, Hatzichristodoulou and collaborators reported [15] that 84% achieved complete penile straightening during immediate follow-up in a previous study. The mean dorsal curvature was 67° (ranging from 30° to 100°), with an average operating time of 94 min (ranging from 65 to 165 min). Notably, 17% of these patients experienced a mild residual curvature of less than ten degrees. An analysis of outcomes from 52 PD patients who underwent CFG after PIG/PEG revealed a 92.3% success in achieving complete curvature correction. Furthermore, 80.8% of patients did not experience significant penile shortening after a 6-month follow-up [17]. The efficacy of this technique has been described in patients with dorsal, dorsolateral, or ventral curvatures [13, 15, 17, 18].

Studies on CFG patch grafting have reported penile straightening rates ranging from 83% to 93.7% [13, 15, 19]. In a retrospective study, Horstmann et al. [13] compared 32 patients undergoing plication (Nesbit or Essed‒Schroeder technique) versus 43 patients who underwent CFG. They reported similar perioperative and postoperative complications in both groups. Satisfaction rates did not differ between the study groups. Overall, 21 patients (66%) with plication and 36 patients (84%) with GFG had a straight or almost straight penis postoperatively. However, patients treated with plication reported better outcomes regarding erectile function, penile length, and sensation. Notably, around 60% of patients expressed their willingness to choose the same intervention again.

Compared to the less invasive character of plication techniques for PD, a decrease in penile sensation (penile hypoesthesia, glans numbness) has been reported in 3% to 31% of the cases with PD after surgery, which can lead to sexual dysfunction [5]. The mobilization of the NVB is often attributed to this typically transient decrease in sensation, with complete resolution reported within 12 months post-surgery in up to 100% of cases [6]. In our study, a decrease in penile sensation was noted in 9% of the patients, with no discernible variance between the different surgical subgroups.

The occurrence of postoperative hematoma, bleeding, and infection remained generally low (2–4.5%). However, one patient experienced postoperative glans necrosis. While this complication has not been reported following plication procedures, it has been documented in up to 2.4% of cases after grafting procedures [6]. Another potential advantage of CFG over other xenografts is its presumed reduced risk of postoperative hematoma due to its hemostatic properties [4, 5, 11].

Recently, Falcone and colleagues compared CFG and porcine small intestine submucosa grafts in patients undergoing plaque incision with grafting and penile prosthesis. After a mean follow-up of 35 months, there were no significant differences in postoperative outcomes. However, the use of CFG was associated with significantly shorter mean operating time (128.8 for TachoSil grafting versus 148.8 for SIS, *p* < 0.0001) and lower costs [20]. The reduced operating time with CFG was linked to a decreased risk of infections [11]. It is important to note that intraoperative assessment of residual penile curvature at maximum erection with intracavernous saline injection is not feasible with CFG due to the risk of graft detachment [4, 17]

Some limitations of the current study need to be acknowledged. The study design was retrospective, thereby constrained by the data routinely collected. Moreover, only early postoperative follow-up assessments were taken into account. Detailed postoperative evaluation of erectile function based on preoperative scores was not documented. The postoperative assessment of erectile function using the IIEF-5 score was not documented for all patients. In addition, patients who received preoperatively PDE5i or intracavernous injections were included, which could introduce confounding factors. The medium-term and long-term outcomes remain undisclosed in our study, potentially revealing significant subgroup disparities in functional outcomes or delayed complications.

## Conclusions

In our retrospective series, early postoperative outcomes and complications seemed similar in patients with mild/moderate vs. severe PD deformities. The technique may represent a viable option for penile straightening in selected cases of PD, particularly when plication is not feasible. Partial plaque excision remains a technically demanding procedure with a notable significant risk of ED and diminished penile sensitivity, irrespective of the type of graft utilized. As such, it should be undertaken exclusively by proficient and high-volume surgeons. A more comprehensive evaluation of postoperative outcomes and long-term follow-up could provide further insights to support the routine implementation of this procedure in clinical practice.

## Data Availability

No datasets were generated or analyzed during the current study.
